# SARS-CoV-2 Pattern Provides a New Scoring System and Predicts the Prognosis and Immune Therapeutic Response in Glioma

**DOI:** 10.3390/cells11243997

**Published:** 2022-12-10

**Authors:** Fan Jiang, Deng-Feng Lu, Zheng Zhan, Gui-Qiang Yuan, Guang-Jie Liu, Jing-Yu Gu, Xiao-Ou Sun, Zhong Wang

**Affiliations:** Department of Neurosurgery & Brain and Nerve Research Laboratory, The First Affiliated Hospital of Soochow University, Suzhou 215006, China

**Keywords:** COVID-19, glioma, immunotherapy, prognosis, molecular mechanism

## Abstract

Objective: Glioma is the most common primary malignancy of the adult central nervous system (CNS), with a poor prognosis and no effective prognostic signature. Since late 2019, the world has been affected by the rapid spread of SARS-CoV-2 infection. Research on SARS-CoV-2 is flourishing; however, its potential mechanistic association with glioma has rarely been reported. The aim of this study was to investigate the potential correlation of SARS-CoV-2-related genes with the occurrence, progression, prognosis, and immunotherapy of gliomas. Methods: SARS-CoV-2-related genes were obtained from the human protein atlas (HPA), while transcriptional data and clinicopathological data were obtained from The Cancer Genome Atlas (TCGA) and Chinese Glioma Genome Atlas (CGGA) databases. Glioma samples were collected from surgeries with the knowledge of patients. Differentially expressed genes were then identified and screened, and seven SARS-CoV-2 related genes were generated by LASSO regression analysis and uni/multi-variate COX analysis. A prognostic SARS-CoV-2-related gene signature (SCRGS) was then constructed based on these seven genes and validated in the TCGA validation cohort and CGGA cohort. Next, a nomogram was established by combining critical clinicopathological data. The correlation between SCRGS and glioma related biological processes was clarified by Gene set enrichment analysis (GSEA). In addition, immune infiltration and immune score, as well as immune checkpoint expression and immune escape, were further analyzed to assess the role of SCRGS in glioma-associated immune landscape and the responsiveness of immunotherapy. Finally, the reliability of SCRGS was verified by quantitative real-time polymerase chain reaction (qRT-PCR) on glioma samples. Results: The prognostic SCRGS contained seven genes, REEP6, CEP112, LARP4B, CWC27, GOLGA2, ATP6AP1, and ERO1B. Patients were divided into high- and low-risk groups according to the median SARS-CoV-2 Index. Overall survival was significantly worse in the high-risk group than in the low-risk group. COX analysis and receiver operating characteristic (ROC) curves demonstrated excellent predictive power for SCRGS for glioma prognosis. In addition, GSEA, immune infiltration, and immune scores indicated that SCRGS could potentially predict the tumor microenvironment, immune infiltration, and immune response in glioma patients. Conclusions: The SCRGS established here can effectively predict the prognosis of glioma patients and provide a potential direction for immunotherapy.

## 1. Introduction

Gliomas are the most common primary malignant tumor of the central nervous system (CNS) in adults, with an annual incidence of 6.6 cases per 100,000 in the United States and 7.0 cases per 100,000 in China [1,2]. As neuroectodermal tumors, gliomas originate from glial or precursor cells and include astrocytomas, glioblastomas, oligodendrogliomas, and ependymomas [3]. According to the fifth edition of the WHO Classification of Tumors of the Central Nervous System (WHO CNS5) in 2021, gliomas are classified from grade II to IV by histological characteristics, together with molecular features such as IDH mutation status, CDKN2A/B deletion status, TERT promoter mutation status and chromosome 1p/19q co-deletion status [4]. Most low-grade gliomas (LGG, WHO Grade II and III) progress to glioblastoma (GBM, WHO Grade IV) in under ten years [5]. About half of newly diagnosed gliomas are classified as glioblastoma, with a median survival of only 12–17 months and a five-year survival rate of less than 5.5% [1]. Despite the comprehensive treatment, including surgery, radiotherapy, chemotherapy, and molecular targeted therapy, the prognosis of gliomas, especially high-grade gliomas, has not been significantly improved [6].

Severe acute respiratory syndrome coronavirus 2 (SARS-CoV-2) is a highly infectious positive-sense, single-stranded RNA virus that is thought to have emerged from a zoonotic source and spread rapidly in humans through respiratory droplets and contact [7]. The rapid global spread of SARS-CoV-2 infection, since late 2019, is known as the coronavirus disease 2019 (COVID-19) pandemic. The COVID-19 pandemic caused by SARS-CoV-2 infection has placed undue strain on health systems due to the threat to population health, particularly those with weakened immunity, such as cancer patients or the elderly [8,9,10,11,12,13,14].

Wang et al. showed that high expression of C1R, chemokine (C-C-motif) ligand 2 (CCL2), and TNF receptor superfamily 1A (TNFRSF1A) in the COVID-19 pathway was significantly associated with low survival in GBM patients and immune cell infiltration, which may be used as molecular biomarkers of prognosis and immune infiltration in patients with GBM in the future [15]. Chen’s team also found that, compared with normal brain tissue, viral receptors Alanyl aminopeptidase (ANPEP) and Glutamyl Aminopeptidase (ENPEP) were significantly increased in both mRNA and protein levels in GBM and were associated with poor prognosis and survival [16]. These studies provide valuable entry points for the further exploration of the correlation between COVID-19 and glioma. This also prompted us to further attempt, through a series of bioinformatics analyses and glioma samples, to better understand the biological characteristics and clinical significance of SARS-CoV-2-related genes in glioma, as well as their potential significance for glioma immunotherapy.

## 2. Materials and Methods

### 2.1. Acquisition of Patient Data

The training set contained 667 glioma patients and 1152 normal people as a control. The RNA-Seq data and corresponding clinicopathological data of these patients were obtained from the TCGA website (http://cancergenome.nih.gov/, accessed on 1 September 2022). A total of 1152 normal brain tissue samples were obtained from Genotype-Tissue Expression Project (GTEX, https://www.gtexportal.org/home/index.html, accessed on 1 September 2022) database.

### 2.2. Identification of SARS-CoV-2 Related Genes for Glioma in TCGA

A total of 333 SARS-Cov-2-related genes were obtained from the human protein atlas (HPA) (https://www.proteinatlas.org/, accessed on 1 September 2022) database. Gene Ontology (GO) and Kyoto Encyclopedia of Genes and Genomes (KEGG) analyses were performed to identify pathways enriched by differentially expressed genes [17]. Then, the mRNA expression and survival data were used to further screen the above genes by univariate COX regression analysis, least absolute shrinkage, selection operator (LASSO) regression, and multivariate COX regression analysis (*p* < 0.05).

### 2.3. Construction of the SARS-CoV-2 Related Risk Signature

The regression coefficients were linearly integrated with the expression levels of seven SARS-CoV-2-related genes to establish the optimal SARS-CoV-2-related risk signature. The risk score is defined here as the SARS-CoV-2 Index (SC2I) and is calculated as follows:

$$\mathrm{SC}2I=\overset{n}{\underset{i=1}{\sum }}\left(\mathrm{Coefi}\times \mathrm{Expi}\right)$$

Expi represents the expression value, and Coefi is the corresponding regression coefficient calculated by multivariate Cox analysis.

### 2.4. Construction and Evaluation of a Predictive Nomogram

Age, sex, MGMT promoter methylation status, IDH mutation status, WHO grade, tumor subtype, and SC2I were combined to construct a nomogram using the R package “survival” and “rms” to predict the probability of clinical events in a personalized way. Calibration curves were adopted to evaluate the accuracy of the nomogram in predicting the 1-, 3-, and 5-year survival rates of glioma patients [18].

### 2.5. Functional Enrichment Analysis

GO enrichment analysis and KEGG pathway analysis were used for functional enrichment analysis via GSEA and R software.

### 2.6. Evaluation of the Immune Landscape

The abundance of 22 immune cells was calculated using the Cibersort algorithm [19]. The extent of 28 infiltrating immune cells was quantified using the single sample gene set enrichment analysis (ssGSEA) of the “gsva” package [20]. Spearman correlation analysis was used to obtain the relationship between risk scores and the expression levels of common immune checkpoints. The ESTIMATE Score, Immune Score, and Stromal Score were calculated using the ESTIMATE algorithm via the R package “estimate” to evaluate the role of SC2I in predicting the glioma tumor microenvironment [21]. Finally, the tumor immune dysfunction and exclusion (TIDE) algorithm (http://tide.dfci.harvard.edu, accessed on 1 September 2022) was applied to investigate the role of SC2I in predicting the response of glioma to immunotherapy.

### 2.7. Validation of SARS-CoV-2 Related Gene Signature in CGGA

Patient data from the CGGA database (http://www.cgga.org.cn/, accessed on 1 September 2022) were used for external validation. The median of the SC2I was used as the boundary to divide the sample into high- and low-risk groups. Different packages in R software were used to draw the Kaplan–Meier curve and ROC curve, and “ggplot2” software was used to draw the PCA scatter plot.

### 2.8. Tissue Samples and Quantitative Real-Time Polymerase Chain Reaction (qRT-PCR)

From May 2022 to August 2022, we collected clinicopathological data, including age, gender, WHO grade, IDH mutation status, MGMT promoter methylation status, and tissue samples of nine patients with glioma, from the First Affiliated Hospital of Soochow University. In this paper, we presented the results of the analysis with WHO grade and IDH mutation status as grouping indicators. This study was approved by the Medical Ethics Committee of the First Affiliated Hospital of Soochow University, and informed consent was obtained from all patients. A quantitative real-time polymerase chain reaction was performed. Tissues were stored at −80 °C, and total RNA was extracted from tissues using RNA Trizol reagent (Beyotime, Shanghai, China) following manufacturer’s instructions. The extracted total RNA was reverse-transcribed into cDNA using reverse transcription reagent, and cDNA was amplified in real-time using SYBR fluorescent quantitative PCR reagent. CT values were measured and calculated by computer software. The relative mRNA levels were normalized to β-actin as an internal control by the 2^−ΔΔCT^ method. Sequences of primers are shown in Supplementary Table S1.

### 2.9. Statistical Analysis

The RNA-seq transcriptome data were preprocessed using Perl programming language (version 5.32.0, created by Larry Wall from Los Angeles, CA, USA). The R software (version 4.2.1, created by Ross Ihaka and Robert Gentlemen from Auckland, New Zealand) was applied for all statistical analyses and graph visualization. The chi-square test was used to compare categorical variables between the high- and low-risk groups. *p* value ≤ 0.05 was considered statistically significant.

## 3. Results

### 3.1. Identification of Prognostic SARS-CoV-2 Related Differentially Expressed Genes in Glioma Patients

**Figure 1:**
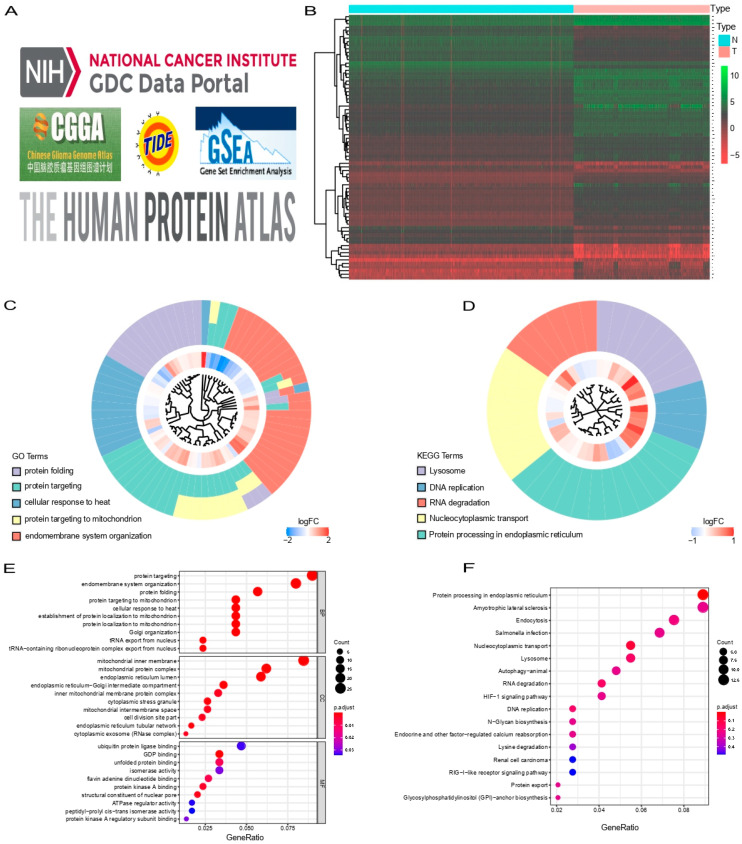
Identification of DEGs and biological characteristics of these genes by GO enrichment analysis and KEGG pathway analysis. (**A**,**B**) Heatmap (**A**) and a volcano plot (**B**) of 74 DEGs in normal and glioma groups. The logFC is presented. (**C**,**E**) Results of GO enrichment analysis. (**D**,**F**) Results of KEGG pathway analysis. The larger the circle, the redder the color and the stronger the correlation. DEGs, differential expressed genes; GO, gene ontology; KEGG, Kyoto encyclopedia of genes and genomes.

**Figure 2:**
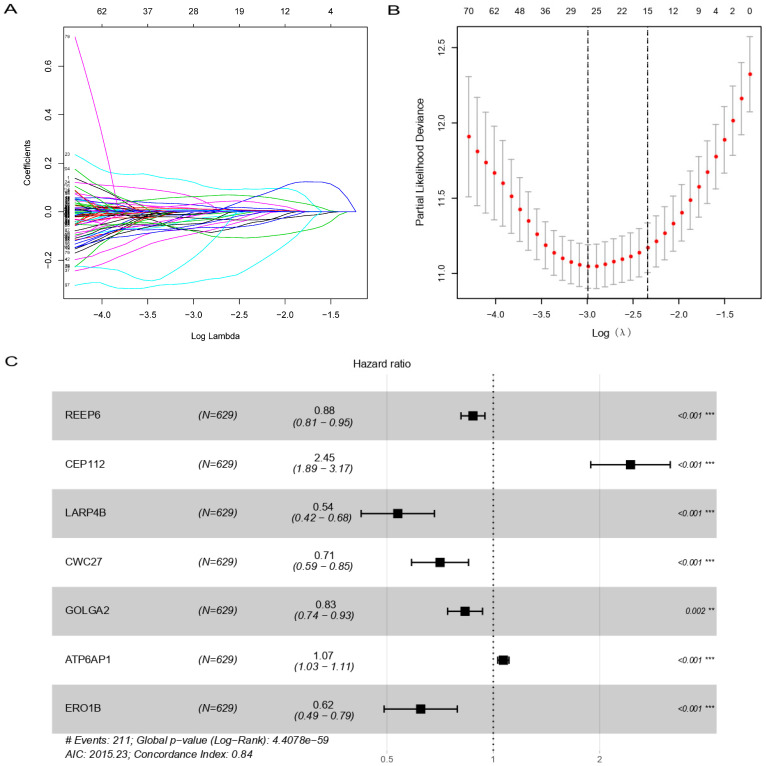
Final acquisition of prognostic DEGs for developing a risk model in TCGA cohort. (**A**) LASSO coefficient profiles of SARS-Cov-2 related genes. (**B**) Partial likelihood deviance of different numbers of variables revealed by the LASSO regression model (The log(λ) sequence plot of SARS-Cov-2 related genes using LASSO regression). (**C**) Forest plot of SARS-Cov-2-related genes. DEGs, differentially expressed genes; LASSO, least absolute shrinkage, and selection operator. ** *p* < 0.01, *** *p* < 0.001.

A total of 74 differential expressed genes (DEGs) were identified by differential analysis between the normal and tumor groups (Figure 1A,B). GO enrichment analysis and KEGG pathway analysis were used to identify the biological characteristics of these DEGs. Protein targeting, protein targeting to mitochondrion, protein folding, endomembrane system organization, and cellular response to heat were identified as the most abundant processes using GO analysis (Figure 1C,E). KEGG pathway analysis suggested that DEGs were significantly enriched in protein processing in the endoplasmic reticulum, nucleocytoplasmic transport, lysosome, RNA degradation, and DNA replication (Figure 1D,F). Thirty-two genes were screened by univariate Cox regression analysis using mRNA expression and survival data from TCGA. These 32 genes were then incorporated into the least absolute shrinkage and selection operator (LASSO) regression to avoid overfitting problems, and 26 genes stood out (Figure 2B,C). Finally, multivariate Cox regression analysis was performed to screen out seven genes, which were REEP6, CEP112, LARP4B, CWC27, GOLGA2, ATP6AP1, and ERO1B (Figure 2D). Pan-cancer analysis of these seven genes mentioned above in the TCGA and GTEX showed variable expression levels in the tumor and normal groups (Supplementary Figure S1). As a result, the identification of prognostic SARS-CoV-2-related genes was completed. Among these genes, CEP112 and ATP6AP1 are risk factors for glioma with hazard ratios (HRs) > 1, while REEP6, LARP4B, CWC27, GOLGA2, and ERO1B are protective factors with HRs < 1. In addition, we explored the proteins expressed by REEP6, LARP4B, CWC27, GOLGA2, ATP6AP1 and ERO1B in glioma patients through the HPA database, and REEP6, LARP4B, GOLGA2 and ATP6AP1 showed higher staining intensity in glioma than in normal brain tissue (Supplementary Figure S2A–F). Statistical histograms are shown in Supplementary Figure S2G.

### 3.2. Construction of Prognostic SARS-CoV-2 Related Gene Signature

The risk score was defined as the SARS-CoV-2 Index (SC2I), which was calculated using the following formula:

SC2I = (−0.132 × REEP6 exp) + (0.895 × CEP112 exp) + (−0.622 × LARP4B exp) + (−0.347 × CWC27 exp) + (−0.184 × GOLGA2 exp) + (0.066 × ATP6AP1 exp) + (−0.473 × ERO1B exp).

### 3.3. Evaluation of SC2I in TCGA

**Figure 3:**
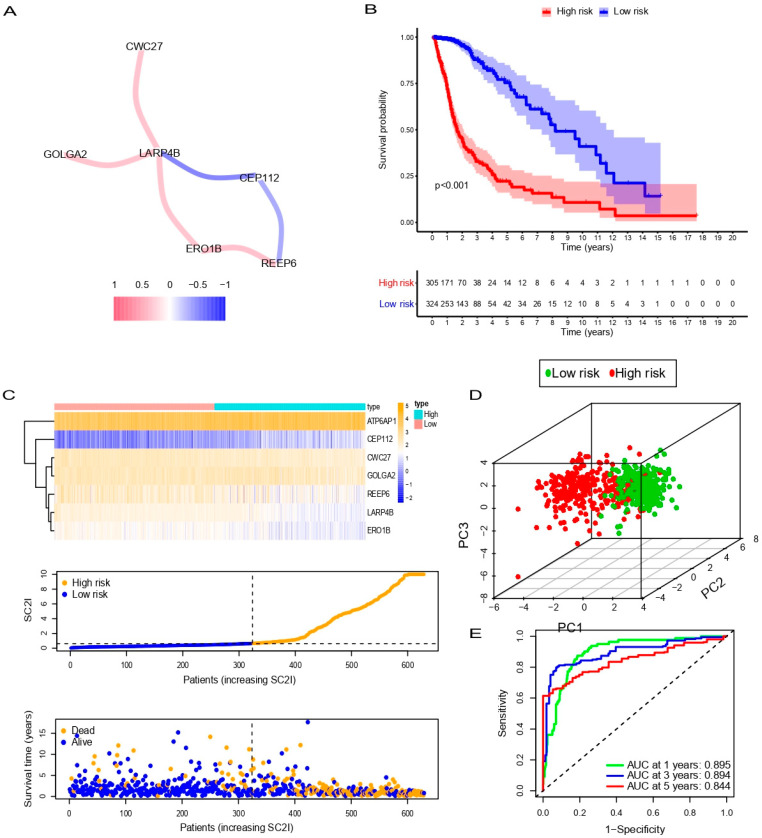
Correlation between SARS-Cov-2-related genes and validation of SCRGS in TCGA cohort. (**A**) Interactions between SARS-Cov-2 related genes. (**B**) Overall survival of different SARS-CoV-2 related genes in glioma. (**C**) Heatmap of SCRGS expression in high- and low-risk groups (upper), distribution and median value of SC2I (middle), and the distributions of survival status, survival time, and SC2I (below). (**D**) PCA analysis of SARS-CoV-2-related gene signature. (**E**) Time-dependent ROC analysis of SARS-CoV-2-related gene signature. SCRGS, SARS-CoV-2-related gene signature; SC2I, SARS-CoV-2 index; PCA, principal component analysis; ROC, receiver operating characteristic.

Then, we analyzed the correlations between the genes from the TCGA dataset using the Pearson correlation analysis. Genes with correlation coefficients >0.3 are shown in Figure 3A, with positive correlations in blue and negative ones in red. The survival curves of each gene are shown in Supplementary Figure S3.

Samples in the TCGA dataset were divided into high-risk and low-risk groups based on the median SC2I. First, we performed a Kaplan–Meier (KM) analysis, which showed that patients in the low-risk group had a higher probability of survival than those in the high-risk group (Figure 3B). The heatmap showed that the expression of the seven modeling genes was differentially expressed in the high- and low-risk groups. Compared with the high-risk group, the expression of CEP112 was decreased in the low-risk group, while the expression level of REEP6, LARP4B, and ERO1B was relatively increased (Figure 3C). Patients in the high-risk group had a shorter survival time than those in the low-risk group (Figure 3C). Then, we performed principal component analysis (PCA) in the TCGA cohort, which showed a significant and stable distribution of differences between the high- and low-risk subgroups (Figure 3D). The time-dependent receiver operating characteristic (ROC) curve was used to evaluate the effectiveness of SC2I in predicting the prognosis of glioma patients. As shown in Figure 3E, the AUC of 1-, 3- and 5-year overall survival (OS) rates in the TCGA dataset were 0.895, 0.894, and 0.844, respectively. These results indicate that the SC2I is accurate in predicting the prognosis of patients with glioma.

### 3.4. Independent Prognostic Value of OS

**Figure 4:**
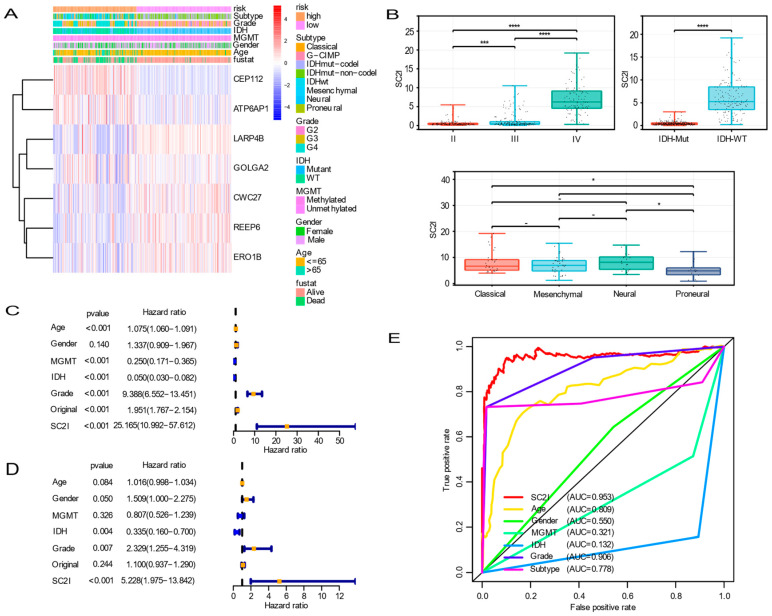
Correlation between SC2I and clinicopathological characteristics in TCGA dataset. (**A**) Heatmap of correlation between risk groups and subtype, WHO grade, IDH mutation status, MGMT promoter status, gender, age, survival status, and mRNA expression of SARS-CoV-2-related gene. (**B**) Different distribution of SC2Is among glioma subgroups (**C**,**D**) Univariate and multivariate COX regression analysis of the combination of SC2I and clinicopathological characteristics. (**E**) ROC curves of SC2I and clinicopathological characteristics. SC2I, SARS-CoV-2 index; TCGA, The Cancer Genome Atlas; IDH, isocitrate dehydrogenase; MGMT, O^6^-methylguanyl DNA methyltransferase; ROC, receiver operating characteristic. * *p* < 0.05, *** *p* < 0.001, **** *p* < 0.0001.

A heatmap containing mRNA expression and other clinical information (subtype, WHO grade, IDH mutation status, MGMT promoter status, gender, age, and survival status) of SARS-CoV-2-related genes showed that the expression of LARP4B, GOLGA2, CWC27, REEP6, and ERO1B were positively correlated with the risk score. In contrast, CEP112 and ATP6AP1 were negatively correlated. Meanwhile, the WHO grade of gliomas was positively correlated with the SC2I, as expected (Figure 4A). SC2I was significantly higher in patients with gliomas of WHO grade IV than those of WHO grade III and II (Figure 4B). An IDH mutation status-based subgroup analysis showed that patients with IDH wild type had significantly higher SC2I than IDH mutated type (Figure 4B). Subgroup analysis based on tumor subtype suggested that the SC2I in the proneuronal type was lower than that in classical and neural type (Figure 4B). The above findings implied that SC2I could differentiate glioma patients classified based on the above key indicators. Univariate Cox and multivariate Cox of SC2I combined with clinical information showed that SC2I itself was an independent risk factor for the prognosis of glioma (Figure 4C,D). Moreover, the ROC curve of SC2I and other clinical characteristics revealed that its predictive performance (AUC = 0.959) was better than other classical indicators, including tumor subtype (AUC = 0.778), WHO grade (AUC = 0.906), IDH mutation status (AUC = 0.132), MGMT promoter status (AUC = 0.321) and age (AUC = 0.809) (Figure 4E).

### 3.5. Establishment of a Nomogram Based on Independent Prognostic Factors for OS and Validation of Its Predictive Accuracy

**Figure 5:**
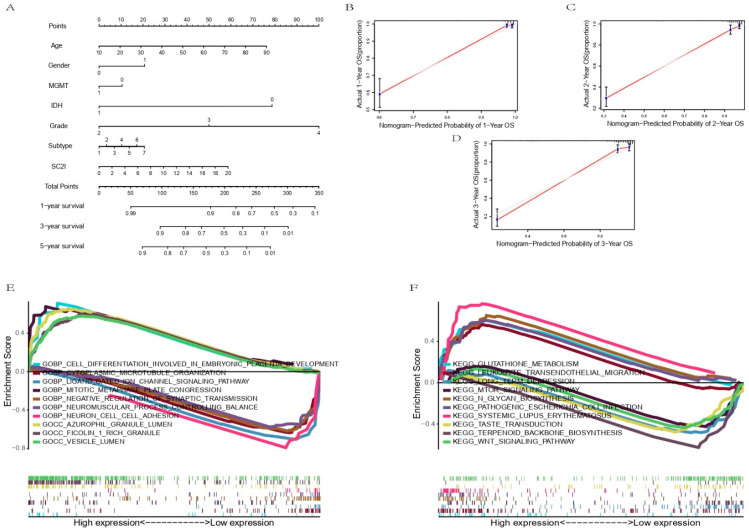
Prediction of the survival of glioma patients by nomogram, and GSEA analysis between different risk groups in TCGA cohort. (**A**) Nomogram used for predicting glioma patients was constructed. (**B**–**D**) Calibration plots for predicting 1-, 2- and 3-year overall survival. (**E**,**F**) GO and KEGG functional enrichment analysis. GSEA, gene set enrichment analysis; TCGA, The Cancer Genome Atlas; GO, gene ontology; KEGG, Kyoto encyclopedia of genes and genomes.

By integrating SC2I, age, gender, MGMT promoter status, IDH mutation status, WHO grade, and original subtype, we established a nomogram to accurately predict the 1-, 2- and 3-year survival probability of glioma patients (Figure 5A). The calibration curves showed significant concordance between the predicted and actual rates of OS at 1, 2, and 3 years in the TCGA dataset (Figure 5B–D).

### 3.6. Functional Enrichment Analysis

Gene set enrichment analysis (GSEA) reminds us of some key pathways. In the seven SARS-Cov-2-related genes, the up-regulated ones are enriched in cell differentiation involved in embryonic placenta development, mitotic metaphase plate congression, azurophil granule lumen, Ficolin-1-rich granule and vesicle lumen by Gene ontology enrichment analysis. Meanwhile, those downregulated genes are rich in the following pathways: neuron cell–cell adhesion, ligand-gated ion channel signaling pathway, cytoplasmic microtubule organization, negative regulation of synaptic transmission, and the neuromuscular process controlling balance (Figure 5E).

The KEGG pathway analysis was also carried out. The results revealed that the upregulated genes are enriched in systemic lupus erythematosus pathways, N-glycan biosynthesis, glutathione metabolism, pathogenic Escherichia coli infection, and leukocyte transendothelial migration. Those down-regulated genes are enriched in terpenoid backbone biosynthesis, long-term depression, taste transduction, WNT signaling pathway, and mTOR signaling pathway (Figure 5F).

### 3.7. Correlation Analysis between Prognostic SCRGS and Immune Status of Glioma

**Figure 6:**
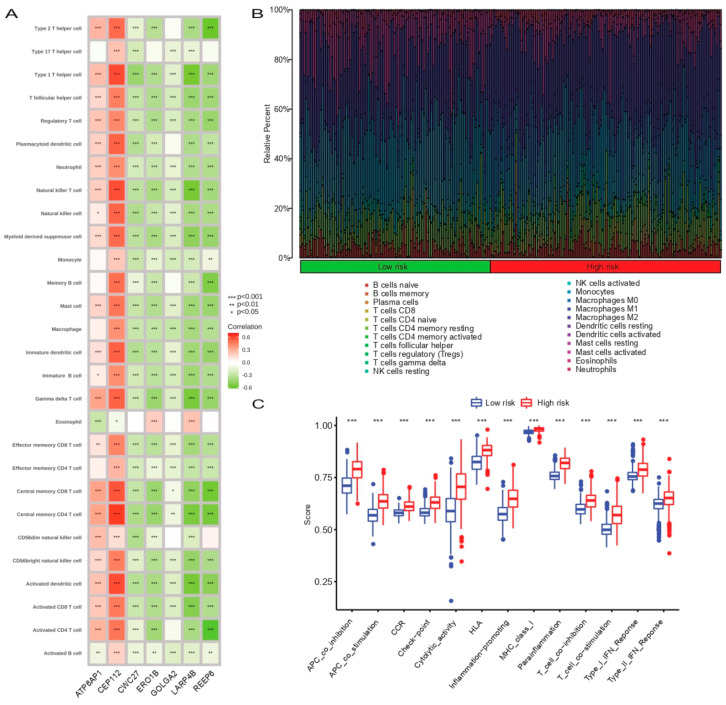
Correlation between the modeling genes and infiltrating immune cells based on ssGSEA. (**A**) Comparison of immune cell infiltration level (**B**) and immune score (**C**) between high- and low-isk groups. ssGSEA, single sample gene set enrichment analysis. * *p* < 0.05, ** *p* < 0.01, *** *p* < 0.001.

We performed ssGSEA analysis to detect the correlation between the modeling genes and 28 types of tumor-infiltrating immune cells. We found that ATP6AP1 and CEP112 had an overall positive correlation with the expression of tumor-infiltrating immune cells, while the five other modeling genes showed the opposite trend (Figure 6A). Based on the immune functional enrichment analysis results, we further explored the correlation between prognostic SCRGS and glioma immune status. The results showed that the infiltration levels of CD8^+^ T cells, follicular helper T cells, M0, M1, and M2 macrophages and neutrophils in the high-risk group were higher than those in the low-risk group. In comparison, the infiltration levels of activated NK cells, monocytes, and activated mast cells were lower than those in the low-risk group. The levels of infiltration of the remaining immune cells were similar between the two groups (Figure 6B). The immune score results showed that the scores of the high-risk group were significantly higher than those of the low-risk group in 13 immune pathways, indicating that anti-cancer immunity was activated (Figure 6C).

**Figure 7:**
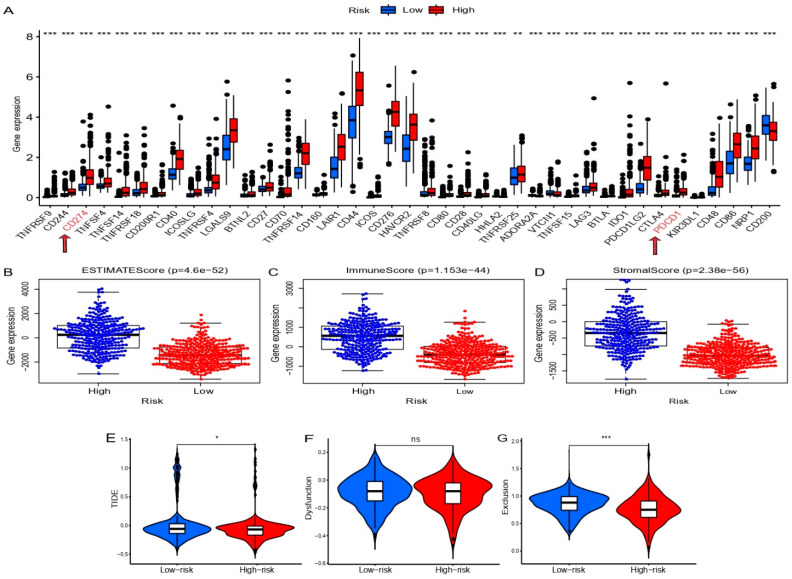
Comparison of immune checkpoint expression levels (**A**), scores for glioma microenvironment (**B**–**D**), and correlation analysis between TIDE evaluation (**E**–**G**) in TCGA dataset between high- and low-risk groups. TIDE, tumor immune dysfunction and exclusion; TCGA, The Cancer Genome Atlas. * *p* < 0.05, ** *p* < 0.01, *** *p* < 0.001, ns, no significance.

In recent years, immune checkpoint blockers (ICB) have made breakthrough progress in the clinical treatment of a variety of malignant tumors [22,23]. Except for CD200, all immune checkpoints were significantly up-regulated in the high-risk group compared with the low-risk group (Figure 7A). CD274 (PD-L1) and PDCD1 (PD-1) are of great importance and play a crucial role in tumor immunosuppression and immunotherapy [24].

In addition, we further evaluated the association between prognostic SCRGS and the glioma microenvironment. ESTIMATE score, immune score, and stromal score in the high-risk group were higher than those in the low-risk group, suggesting a potential correlation between SCRGS and the tumor microenvironment of gliomas (Figure 7B–D). We analyzed TIDE scores in both groups to further clarify the value of prognostic SCRGS in predicting response to immunotherapy. The high-risk group had lower TIDE and exclusion scores (Figure 7E,F), while the dysfunction scores were similar between the two groups (Figure 7G). These results suggest that patients in the high-risk group are more sensitive to immunotherapy. In conclusion, SCRGS can predict immunotherapy response in patients with glioma to some extent.

### 3.8. Validation of the Prognostic Model in the CGGA Cohort

**Figure 8:**
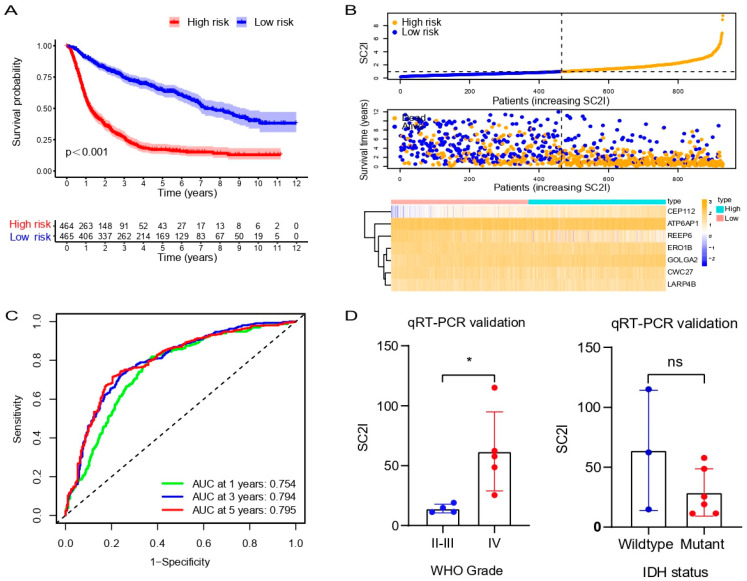
Validation of SCRGS in CGGA dataset. (**A**) Survival curve of SCRGS risk model in high- and low-risk groups. (**B**) Patients in the CGGA dataset were divided into high- and low-risk groups according to the increasing SC2I (**upper**). The distributions of survival status, survival time, and SC2I (**middle**). Heatmap of the modeling genes expression in high- and low-risk groups (**below**). (**C**) Time-dependent ROC analysis of SCRGS. (**D**) QRT-PCR validation of SC2I between WHO II-III grade and WHO IV grade groups (left), and IDH wildtype and IDH mutant groups. SCRGS, SARS-CoV-2-related gene signature; CGGA, Chinese Glioma Genome Atlas; SC2I, SARS-CoV-2 index; ROC, receiver operating characteristic. qRT-PCR, quantitative real-time polymerase chain reaction; SC2I, SARS-CoV-2 index; IDH, isocitrate dehydrogenase. * *p* < 0.05; ns, no significance.

To confirm the reliability of SCRGS, we performed an external validation in CGGA. Firstly, 929 glioma patients in CGGA were divided into a high-risk group and a low-risk group based on the median risk score (Figure 8B). It was found that the survival probability and time of the high-risk group were significantly lower than those of the low-risk group (Figure 8A,B). Next, ROC curve analysis was then performed to further verify the accuracy of prognostic SCRGS in predicting the prognosis of patients with glioma in CGGA. The AUC of 1, 3 and 5 years is 0.754, 0.794 and 0.795, respectively (Figure 8C). The expression of seven modeling genes in the validation cohort was also detected. It is not difficult to conclude that the expression of these genes in the CGGA cohort was consistent with that in the TCGA cohort (Figure 8B). These were all consistent with the results in the training set. Above all, these results indicate that the SARS-CoV-2-related gene signature constructed using the TCGA dataset has a good predictive effect regarding the prognosis of glioma patients.

### 3.9. Validation of Prognostic Model in Glioma Samples

To validate the predictive power of SCRGS in glioma samples, a qRT-PCR analysis of the collected glioma samples was performed. These samples were first divided into WHO grade II-III (4 cases) and grade IV (5 cases), and the SC2I scores were calculated for both groups. It was found that the SC2I of the WHO grade IV group was significantly higher than the corresponding score of the WHO grade II-III group (*p* = 0.025), which was consistent with our expectation (Figure 8D, left). Then, the tumor samples were divided into wild-type and mutant groups according to IDH mutation status. The results showed that there was no significant difference in SC2I scores between the two groups (Figure 8D, right).

## 4. Discussion

Glioma is one of the most common primary central nervous system malignancies, with a poor prognosis and no effective prognostic indicators. HPA has been making progress in fighting the COVID-19 pandemic, and a list of genes related to SARS-CoV-2 based on transcriptomics and antibody-based proteomics was formed. The entry of SARS-CoV-2 into human cells depends on the SARS-CoV-2 receptor, angiotensin-converting enzyme 2 (ACE2) receptor, and cathepsin. Cathepsin degrades the spike protein (S protein), causing viral nucleic acid to enter human host cells. ACE2 is highly expressed in endothelial cells (ECs), bone marrow mesenchymal stem cells (BMSCs), and neural progenitor cells (NPCs), which further differentiate into neurons and glial cells [25,26]. Given this, the nervous system is highly susceptible to SARS-Cov-2. Cathepsin B(CTSB) and cathepsin L (CTSL) is also strongly expressed in various cell clusters in the glioblastoma microenvironment [27]. Furthermore, the expression level of CTSL in GBM tissue was higher than that in normal brain tissue, which was associated with the significantly reduced survival rate of GBM patients. Meanwhile, the expression level of CTSL was negatively correlated with the purity, B cells, and CD8^+^ T cells in GBM, suggesting that CTSL, an independent prognostic factor, can be considered a promising therapeutic target [28]. Further research also demonstrated the sensitivity of neurons and glial cell lines to SARS-CoV-2 infection at high multiplicity of infection [29]. The S protein is a key molecule for virus entry into host cells through interactions with ACE2 receptor molecules on cell membranes. Research by Khan et al. indicated that the S protein has a high affinity to epidermal growth factor receptors (EGFR) and vascular endothelial growth factor receptors (VEGFR)-overexpressing receptors in glioma cells, and may play a role in glioma tumorigenesis [30]. Thus, SARS-Cov-2 is closely associated with the occurrence, progression, and immune microenvironment of gliomas through several mechanisms, which ignited our interest in conducting related studies.

In this study, 333 SARS-Cov-2-related genes were obtained from the human protein atlas. After differential analysis, univariate Cox regression, lasso regression, and multivariate Cox regression, seven genes were finally obtained: REEP6, CEP112, LARP4B, CWC27, GOLGA2, ATP6AP1, and ERO1B.

The two previously reported gliomas of these seven genes are La-related protein 4b (LARP4B) and ATPase H^+^ Transporting Accessory Protein 1 (ATP6AP1). LARP4B is a member of the evolutionarily conserved RNA-binding protein family, which is characterized by the La module involved in direct RNA binding [31]. LARP4B expression was reported to be consistently decreased in human glioma stem cells and cell lines compared with normal neural stem cells. The heterozygous deletion of LARP4B was detected in nearly 80% of glioblastomas in the TCGA database, which was associated with poor patient survival. The overexpression of LARP4B strongly inhibited cell proliferation in glioma by inducing mitotic arrest and apoptosis, which was partly dependent on p53 activity and the La module responsible for the RNA chaperone activity. LARP4B depletion in primary astrocytes from p53 and Nf1-deficient mice promoted cell proliferation and increased gliomas’ size and invasiveness [32]. These data strongly suggest that LARP4B acts as a tumor-suppressor gene of gliomas, as confirmed in our work, as LARP4B expression was negatively correlated with SC2I. Interestingly, there were also reports of LARP4B helping to predict prognosis and immunotherapy in glioma patients [33,34]. In summary, LARP4B is a tumor-suppressor gene of glioma; the potential mechanisms involved, however, remain to be probed further. The ATPase H^+^ Transporting Accessory Protein 1 (ATP6AP1) is a vacuolar ATPase proton pump component [35]. ATP6AP1 gene mutations are involved in congenital disorders of glycosylation (CDG) and affect multiple organ systems, including immunodeficiency and cognitive impairment [36,37,38]. One study recently showed that a risk signature including ATP6AP1 could reasonably predict the prognosis and immune microenvironment of glioma patients [39]. This corroborates the findings of our work to some extent. Non-structural protein 6 (NSP6) of SARS-Cov-2 was reported to induce NLRP3-dependent apoptosis in lung epithelial cells, with ATP6AP1 playing a pivotal role [35]. More experimental studies of LARP4B and ATP6AP1 in glioma are urgently needed to clarify their specific functions and potential mechanisms.

The remaining five genes have not been reported in glioma, but it is necessary to recognize them to provide some reference for follow-up work. One article from India suggested that the mutual interaction between Centrosomal Protein 112 (CEP112) and breast cancer type 1 susceptibility protein (Brca1) was significant in the mitotic regulation and maintenance of genomic stability [40]. CWC27 is one of the known human cyclophilins, also known as SDCCAG10, recruited by the spliceosome for the pre-mRNA splicing process [41]. A study by Wang et al. showed that CWC27 plays an oncogenic role in bladder cancer by inducing cell proliferation and inhibiting cell apoptosis [42]. GOGLA2 encodes GM130, a Golgi protein involved in vesicle binding, cell proliferation, and autophagy [43,44,45]. In two earlier studies, GOLGA2 downregulation inhibited tumorigenesis and cell invasion in gastric and lung cancers, suggesting that GOLGA2 appears to be an oncogene [46,47]. Endoplasmic reticulum oxidoreductin 1B (ERO1B) is a pancreas-specific disulfide oxidase that promotes insulin biogenesis in pancreatic β-cells, thus contributing to physiological glucose homeostasis [48,49]. Asada et al. suggested that ERO1B expression levels were associated with lung adenocarcinoma (LUAD) patient survival [50]. Another study showed ERO1B was one of the prognostic indicators of esophageal cancer (EC) [51]. The Receptor Expression-enhancing Protein 6 (REEP6), a member of the Yop1/Yip family, is associated with the enhancement of cell surface expressions and endoplasmic reticulum membrane shaping [52]. It has not been reported in tumors to date. We expect that these genes will be intensively studied in gliomas over the near future, which will greatly benefit our insight into the potential correlation between gliomas and SARS-CoV-2.

Based on these differentially expressed genes, we constructed a risk score, SC2I, and divided glioma patients in the training cohort into high- and low-risk groups according to the median value of SC2I. There were significant differences in gene expression pattern, survival probability, and survival time between the two groups. ROC curve also showed that SC2I made a good prediction of survival rate in patients with glioma. Univariate and multivariate COX regression analysis showed that SC2I was an independent risk factor for glioma prognosis. The ROC curve further showed that SC2I had higher prediction accuracy than the WHO grade. Combining these clinicopathological features, we constructed a nomogram with an improved ability to predict overall survival. Functional enrichment analysis showed that there were differences in cellular biological processes between high- and low-risk groups. The differences in tumor immune status between the two groups were further analyzed. SC2I was positively correlated with immune cell infiltration and immune pathway activation level. The immune checkpoints, including CD274 and PDCD1, were significantly differentially expressed between the two groups, and the TIDE score and exclusion score were significantly different between the two groups, which provides a preliminary basis for glioma immunotherapy. The above work provides a potentially more accurate method for the diagnosis and prognosis prediction of glioma. It provides a certain theoretical basis for future immunotherapy, which is expected to be further verified and applied in the near future.

Based on the above identified genes, we constructed a risk score, SC2I, and divided glioma patients in the training cohort into high and low-risk groups according to the median value of SC2I. There were significant differences in gene expression pattern, survival probability, and survival time between the two groups. The ROC curve also showed that SC2I had a good prediction of survival rate in patients with glioma. Univariate and multivariate COX regression analysis showed that SC2I was an independent risk factor for glioma prognosis. The ROC curve further showed that SC2I had higher prediction accuracy than the WHO grade. Combining these clinicopathological features, we constructed a nomogram with an improved ability to predict overall survival. Functional enrichment analysis showed that there were differences in cellular biological processes between high- and low-risk groups. The differences in tumor immune status between the two groups were further analyzed. SC2I was positively correlated with immune cell infiltration and immune pathway activation level. The immune checkpoints, including CD274 and PDCD1, were significantly differentially expressed between the two groups, and the TIDE score and exclusion score were significantly different between the two groups, which provides a preliminary basis for immunotherapy of glioma.

This study still has some limitations. First, our current results were mainly from TCGA and CGGA databases. Since TCGA is underrepresented in terms of sample size and ethnic differences, further validation in more glioma cohorts is warranted. Second, the number of qRT-PCR samples used for validation was insufficient. It is necessary to collect more tumor samples to verify the reliability of the model. In addition, the functions and specific mechanisms of these genes in glioma are still unclear and need to be further explored.

## 5. Conclusions

In conclusion, we identified seven SARS-Cov-2-related genes using differential analysis, COX regression and LASSO regression analysis. Then, we established a glioma prognostic model based on the above genes. This model can predict the prognosis of glioma patients and is correlated with the immune landscape of glioma microenvironment. As a result, our findings offer some valuable insights into future research and clinical practice regarding gliomas.

## Data Availability

Public data were from https://www.gtexportal.org/home/index.html (GTEX), http://cancergenome.nih.gov/ (TCGA), http://www.cgga.org.cn/ (CGGA), https://www.proteinatlas.org/ (HPA), http://tide.dfci.harvard.edu (TIDE), accessed on 1 September 2022.

## Supplementary Materials

The following supporting information can be downloaded at: https://www.mdpi.com/article/10.3390/cells11243997/s1, Figure S1. The expression of the seven SCRGs. The differential expression of (A) REEP6, (B) CEP112, (C) LARP4B, (D) CWC27, (E) GOLGA2, (F) ATP6AP1 and (G) ERO1B in 33 tumour types based on TCGA and GTEx cohorts. (* *p* < 0.05, ** *p* < 0.01, *** *p* < 0.001. **** *p* < 0.0001); Figure S2. Representative images of immunohistochemistry of the modeling genes (A–F) from HPA and the corresponding statistical plot (G). HPA, human protein atlas; Figure S3. Survival curve of SARS-CoV-2 related genes in TCGA dataset. TCGA, The Cancer Genome Atlas; Table S1. Primer sequences for qRT-PCR validation of SARS-CoV-2 related genes in glioma samples. qRT-PCR, quantitative real-time polymerase chain reaction.
