# Supplementary material for: SARS-CoV-2 Pattern Provides a New Scoring System and Predicts the Prognosis and Immune Therapeutic Response in Glioma
Source: Cells. 2022 Dec 10;11(24):3997. doi: 10.3390/cells11243997 (PMC9777143; doi:10.3390/cells11243997)
Supplement: Supplementary file 1 [file cells-11-03997-s001.zip › cells- 2035389-supplementary materials.pdf]

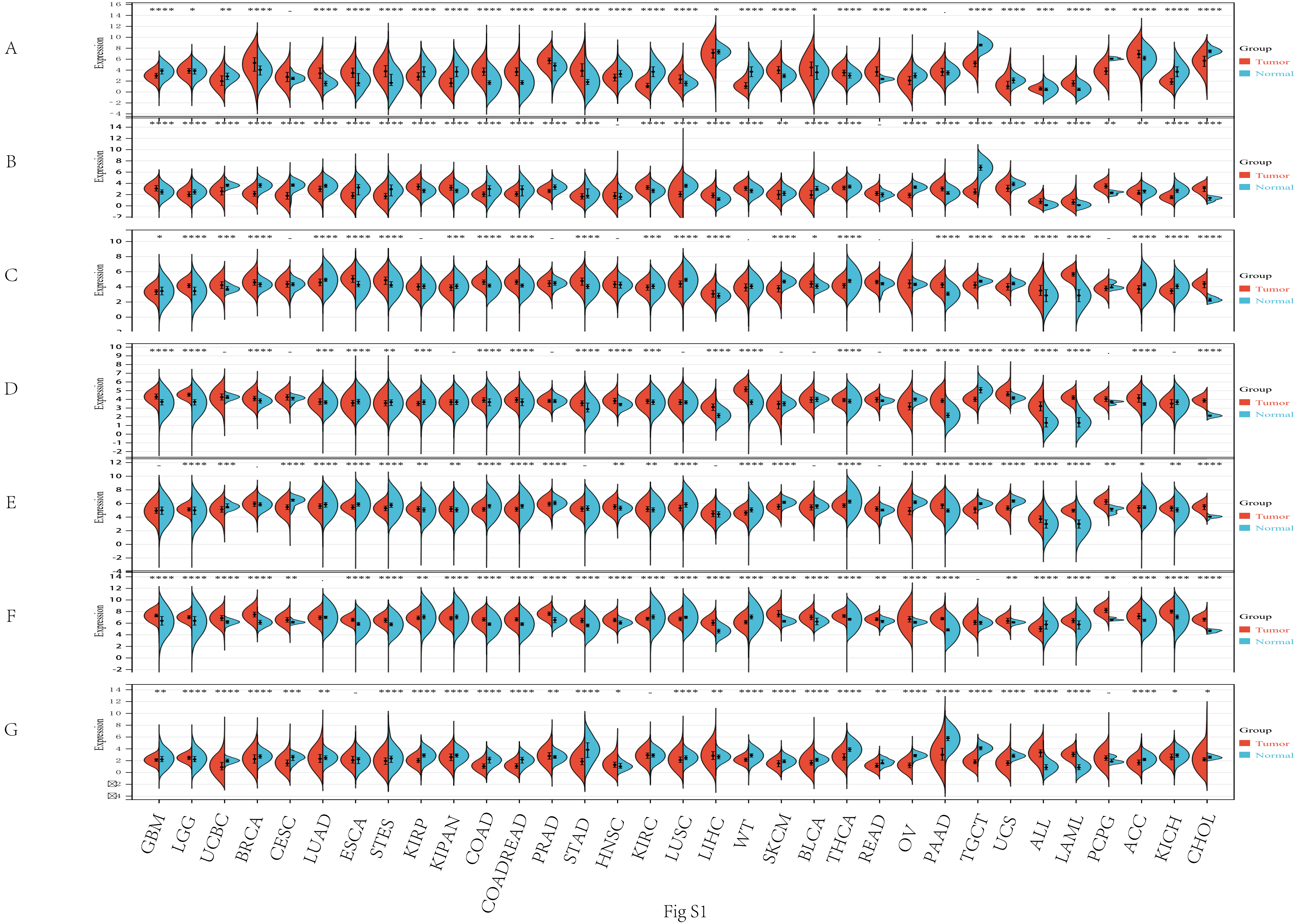

A
B
C
D
E
F
G
Expression
Group
Tumor
Normal
GBM
LGG
UCBC
BRCA
CESC
LUAD
ESCA
STES
KIRP
KIPAN
COAD
COADREAD
PRAD
STAD
HNSC
KIRC
LUSC
LIHC
WT
SKCM
BLCA
THCA
READ
OV
PAAD
TGCT
UCS
ALL
LAML
PCPG
ACC
KICH
CHOL

Fig S1

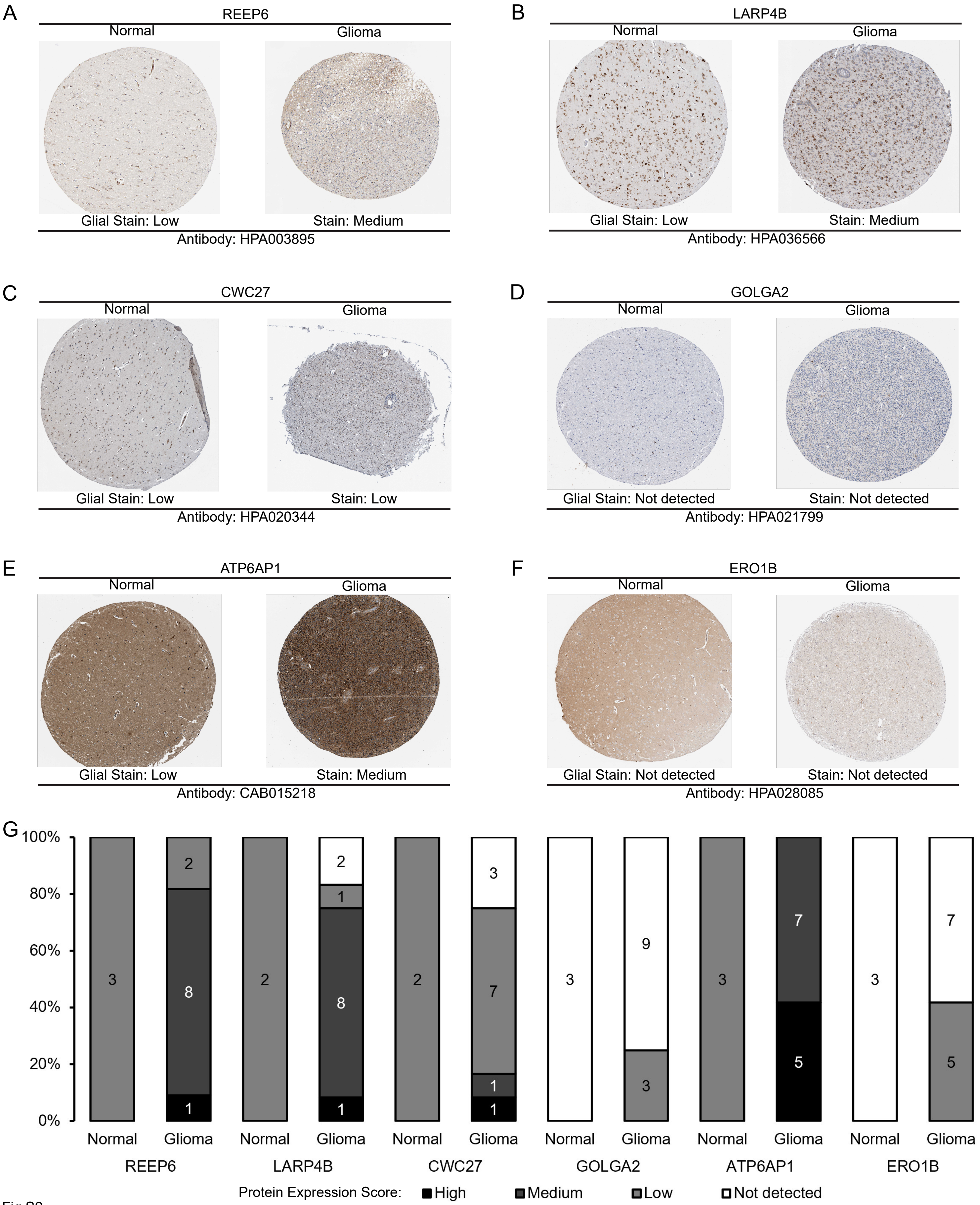
A
REEP6
Normal
Glioma
Glial Stain: Low
Stain: Medium
Antibody: HPA003895
B
LARP4B
Normal
Glioma
Glial Stain: Low
Stain: Medium
Antibody: HPA036566
C
CWC27
Normal
Glioma
Glial Stain: Low
Stain: Low
Antibody: HPA020344
D
GOLGA2
Normal
Glioma
Glial Stain: Not detected
Stain: Not detected
Antibody: HPA021799
E
ATP6AP1
Normal
Glioma
Glial Stain: Low
Stain: Medium
Antibody: CAB015218
F
ERO1B
Normal
Glioma
Glial Stain: Not detected
Stain: Not detected
Antibody: HPA028085
G
100%
80%
60%
40%
20%
0%
3
2
8
1
2
2
1
8
1
2
3
7
1
1
3
9
3
3
7
5
3
7
5
Normal
Glioma
REEP6
LARP4B
CWC27
GOLGA2
ATP6AP1
ERO1B
Protein Expression Score:
High
Medium
Low
Not detected

Fig S2

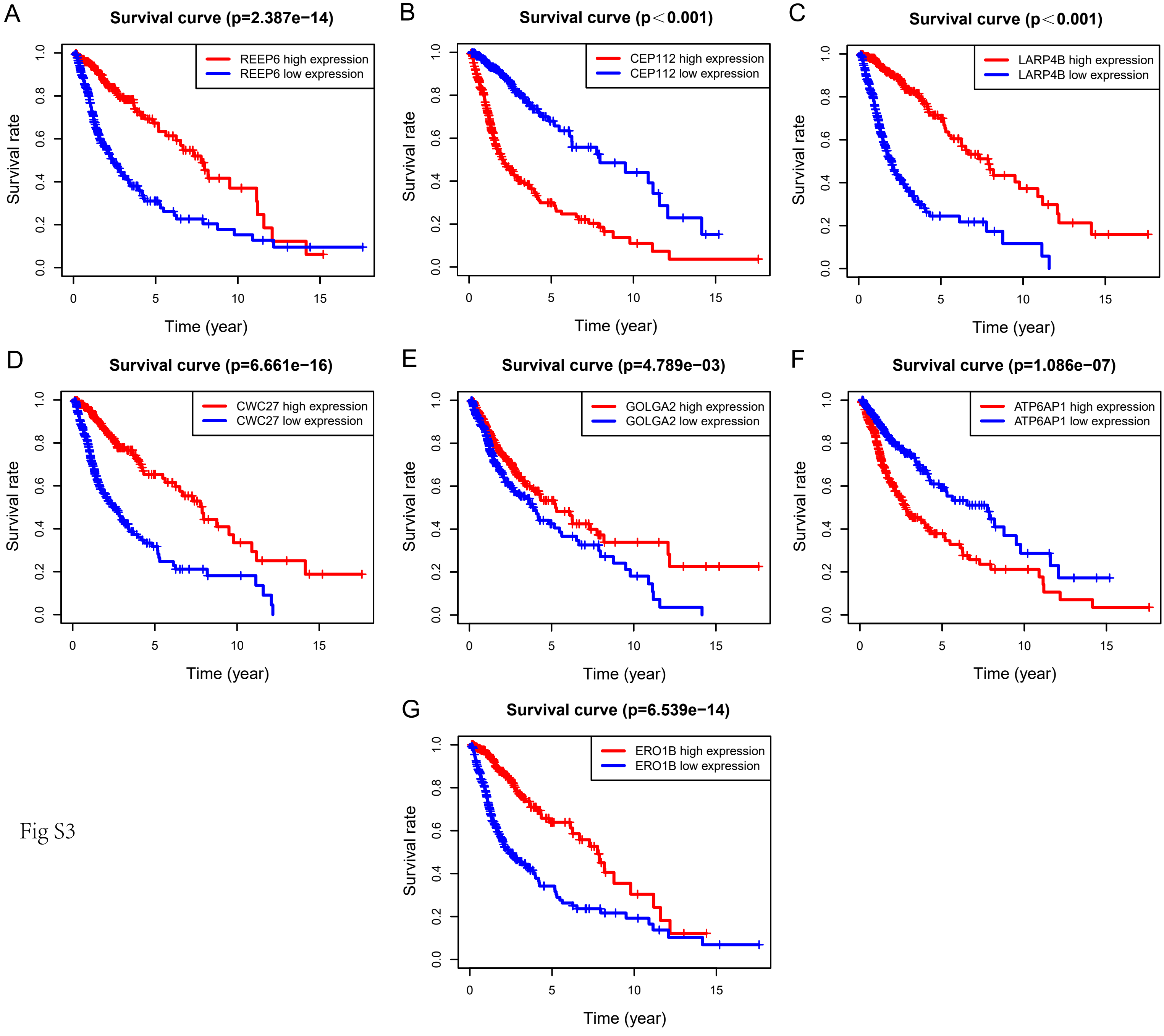
A
Survival curve (p=2.387e−14)
REEP6 high expression
REEP6 low expression
B
Survival curve (p＜0.001)
CEP112 high expression
CEP112 low expression
C
Survival curve (p＜0.001)
LARP4B high expression
LARP4B low expression
D
Survival curve (p=6.661e−16)
CWC27 high expression
CWC27 low expression
E
Survival curve (p=4.789e−03)
GOLGA2 high expression
GOLGA2 low expression
F
Survival curve (p=1.086e−07)
ATP6AP1 high expression
ATP6AP1 low expression
G
Survival curve (p=6.539e−14)
ERO1B high expression
ERO1B low expression
Survival rate
Time (year)
0.0
0.2
0.4
0.6
0.8
1.0
0
5
10
15

Fig S3

Table S1. Sequences of primers

Gene	Primer	Sequence
REEP6	Forwards	GGGTGGAGAAGCGGTATCTG
	Reverse	TCCGATGAGATTGCACAGCAG
CEP112	Forwards	GGCGGCTCCAGGATGTTAG
	Reverse	CAAGTTCTTGTCTTAGGGATGCT
LARP4B	Forwards	AGGACGCTAAGGTTGTGGC
	Reverse	GGTGGGATGGAACTTGTCTGA
CWC27	Forwards	GCCTGGTTTCATAGTCCAAGG
	Reverse	TGAATGGCGCTCCATAGATAGA
GOLGA2	Forwards	CCCGCGATGTCGGAAGAAA
	Reverse	GCATTGTCCTTGGGTGTATCCT
ATP6AP1	Forwards	CAGCGACTTGCAGCTCTCTAC
	Reverse	TGAAATCCTCAATGCTCAGCTTG
ERO1B	Forwards	TTCTGGATGATTGCTTGTGTGAT
	Reverse	GGTCGCTTCAGATTAACCTTGT
